# microRNA-455 targets cullin 3 to activate Nrf2 signaling and protect human osteoblasts from hydrogen peroxide

**DOI:** 10.18632/oncotarget.19486

**Published:** 2017-07-22

**Authors:** Dawei Xu, Hao Zhu, Chengniu Wang, Xinhui Zhu, Genxiang Liu, Chu Chen, Zhiming Cui

**Affiliations:** ^1^ Department of Orthopaedics, The Second Affiliated Hospital of Nantong University, Nantong, China; ^2^ Department of Orthopaedics, The Fourth Affiliated Hospital of Nantong University, Yancheng, China; ^3^ Basic Medical Research Centre, Medical College, Nantong University, Nantong, China

**Keywords:** osteoblast, hydrogen peroxide (H_2_O_2_), Nrf2, microRNA-455, cullin 3 (Cul3)

## Abstract

Over-production of hydrogen peroxide (H_2_O_2_) will lead to human osteoblast dysfunction and apoptosis, causing progression of osteoporosis and osteonecrosis. NF-E2-related factor 2 (Nrf2) is a well-characterized anti-oxidant signaling. Cullin 3 (Cul3) ubiquitin E3 ligase dictates Nrf2 degradation. We demonstrate that microRNA-455 (“miR-455”) is a putative Cul3-targeting microRNA. Forced-expression of miR-455 in both hFOB1. 19 osteoblast cell line and primary human osteoblasts induced Cul3 degradation and Nrf2 protein stabilization, which led to subsequent transcription of ARE (anti-oxidant response element)-dependent genes (*NQO1*, *HO1* and *GCLC*). Cul3 silencing, by expressing miR-455 or targeted-shRNA, protected human osteoblasts from H_2_O_2_. Reversely, miR-455 anti-sense caused Cul3 accumulation and Nrf2 degradation, which exacerbated H_2_O_2_ damages in hFOB1. 19 cells. Moreover, forced over-expression of Cul3 in hFOB1. 19 cells silenced Nrf2 and sensitized H_2_O_2_. Together, we propose that miR-455 activated Nrf2 signaling and protected human osteoblasts from oxidative stress possibly via targeting Cul3.

## INTRODUCTION

Over-production of reactive oxygen species (ROS) shall cause oxidative damages to human osteoblasts. It is the key contributor of osteoporosis or even osteonecrosis [1–4]. Hydrogen peroxide (H_2_O_2_) is one primary ROS [5, 6]. H_2_O_2_ elevation causes profound oxidative stress, osteoblast dysfunction and apoptosis [7–10]. *In vitro* studies have been adding H_2_O_2_ to the cultured human osteoblasts to establish a cellular model of osteoporosis/osteonecrosis [7–10]. This model would help to understand the pathological mechanisms of ROS-induced osteoblast injuries, and to develop possible intervention strategies [11–16].

Nrf2 (NF-E2-related factor 2)-ARE (anti-oxidant response element) pathway is principally mediated by the ubiquitin proteasome system [17–22]. In the resting state, Nrf2 forms a complex with its repressor protein Keap1 (Kelch-like erythroid cell-derived protein with CNC homology [ECH]-associated protein 1) and ubiquitin E3 ligase cullin 3 (Cul3). This leads to Nrf2 protein degradation via ubiquitin-mediated proteolysis [17–22]. Conversely, Nrf2 activation will result in impairment of the Nrf2 ubiquitination and degradation [23–25]. That will allow Nrf2 stabilization, accumulation and translocation to the nucleus, where it transcriptionally activates targeted anti-oxidant genes [17–22].

MicroRNAs (miRs) bind to 3’-untranslated region (UTR) of targeted-mRNAs, thereby causing mRNA degradation and/or the translation inhibition [26, 27]. miRs could be a novel and promising strategy to activate Keap1-Nrf2 signaling [28, 29]. It has been shown that miR-7 targeted Keap1, leading to Nrf2 protein stabilization and subsequent heme oxygenase-1 (HO1) expression [30]. Similarly, miR-141 activated Nrf2 signaling via silencing Keap1 [31]. Meanwhile, miR-141-activated Nrf2 signaling also protected human retinal pigment epithelium cells and retinal ganglion cells from UV radiation [29]. Further, miR-200a expression resulted in Keap1 degradation, leading to Nrf2 nuclear translocation and expression of anti-oxidant gene NADPH quinone oxidoreductase 1 (NQO1) [32].

Here, we identified microRNA-455 (miR-455) as a putative Cul3-targeting miRNA. More importantly, forced-expression of miR-455 activated Nrf2 signaling possibly via silencing Cul3, which protected human osteoblasts from H_2_O_2_.

## RESULTS

### miR-455 expression silences Cul3, causing Nrf2 protein stabilization in human osteoblastic cells

**Figure 1:**
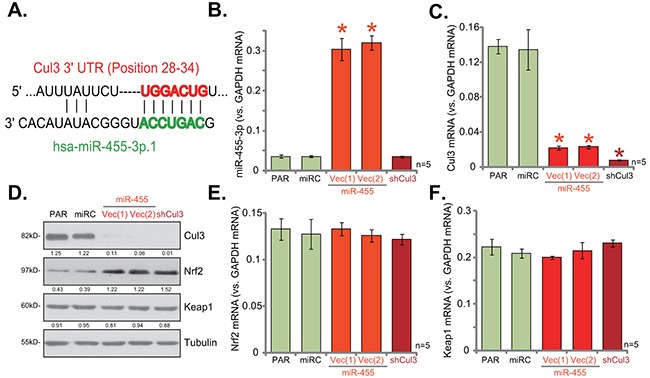
miR-455 expression silences Cul3, causing Nrf2 protein stabilization in human osteoblastic cells miR-455 (3p) targets the 3-UTR of Cul3 mRNA at position 28-34 **(A)**. Stable hFOB1. 19 osteoblastic cells (puromycin-selected), expressing miRNA-455 Vector [two lines, “Vec (1)/(2)”], microRNA-control (“miRC”) or the Cul3-shRNA (“shCul3”), as well as the parental control hFOB1. 19 cells (“PAR”) were subjected to qRT-PCR assay (**B**, **C**, **E** and **F**) and Western blotting assay **(D)** of listed miRNA and genes. Expression of listed proteins was quantified, and was normalized to loading control Tubulin (D). Data were shown as mean (n=5) ± standard deviation (SD). *p<0.05 vs. “PAR” cells. Experiments in this figure were repeated four times, and similar results were obtained.

First, the miRNA database TargetScan was consulted, and potential Cul3-targeting miRNA was searched. We discovered that miR-455 (“-3p.1”) putatively targets the 3-UTR of Cul3 mRNA at position 28-34 (Figure 1A). Thereafter, a miR-455-expressing vector (pSuper-GFP-puro) was constructed (See Method), which was introduced to hFOB1. 19 human osteoblastic cells. Via puromycin selection, two stable hFOB1. 19 cell lines with the construct, namely miR-455 Vec (1)/(2), were established. As shown in Figure 1B, miR-455 (-3p) expression level was significantly increased in the stable cells. Remarkably, miR-455 expression dramatically decreased Cul3 mRNA expression in hFOB1. 19 cells (Figure 1C). Moreover, Cul3 protein was also downregulated in miR-455-expressing cells (Figure 1D). Consequently, Nrf2 protein (Figure 1D), but not Nrf2 mRNA (Figure 1E), was upregulated, indicating Nrf2 protein stabilization. Notably, Keap1 protein (Figure 1D) and mRNA (Figure 1F) were unchanged after miR-455 expression. The microRNA-control (“miRC”) (Figure 1B), as expected, had no significant effect on expression of Nrf2, Keap1 nor Cul3 (Figure 1C-1F). These results suggest that expression of miR-455 targets and downregulates Cul3, causing Nrf2 protein stabilization.

If miR-455 expression targets Cul3 to induce Nrf2 accumulation, Cul3 knockdown should also stabilize Nrf2. Thus, the lentiviral Cul3-shRNA was utilized. Expression of Cul3-shRNA (“shCul3”) almost completely depleted Cul3 mRNA (Figure 1C) and protein (Figure 1D) in hFOB1. 19 cells. Similarly, Nrf2 protein was significantly increased (Figure 1D). Yet, Nrf2 mRNA (Figure 1E) and Keap1 mRNA (Figure 1F) expressions were not changed by the Cul3-shRNA. Thus, Cul3 silence causes Nrf2 stabilization in hFOB1. 19 cells.

### Cul3 knockdown by expressing miR-455 or targeted-shRNA protects hFOB1. 19 cells from H_2_O_2_

**Figure 2:**
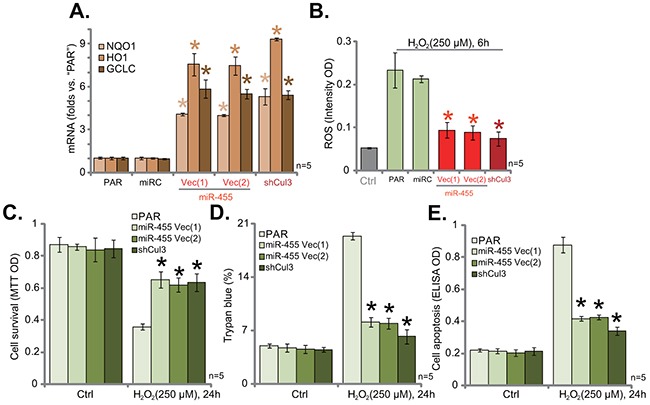
Cul3 knockdown by expressing miR-455 or targeted-shRNA protects hFOB1 19 cells from H_2_O_2_. Stable hFOB1. 19 cells (puromycin-selected), expressing miRNA-455 Vector [two lines, “Vec (1)/(2)”], microRNA-control (“miRC”) or the Cul3-shRNA (“shCul3”), as well as the parental control hFOB1. 19 cells (“PAR”) were treated with/out H_2_O_2_ (250 μM, for applied time), relative NQO1, HO1 and GCLC mRNA expressions were tested by qRT-PCR assay **(A)**. Cellular ROS intensity was tested by DCFH-DA fluorescent dye assay **(B)**. Cell viability, cell death and apoptosis were tested by MTT assay **(C)**, Trypan blue staining assay **(D)** and histone DNA ELISA assay **(E)**, respectively. Data were shown as mean (n=5) ± standard deviation (SD). *p<0.05 vs. “PAR” cells. Experiments in this figure were repeated three times, and similar results were obtained.

The results above demonstrated that Cul3 depletion, by expressing miR-455 or targeted-shRNA, caused Nrf2 protein stabilization in hFOB1. 19 cells. Nrf2, once stabilized, shall translocate to nuclei and dictate transcription of anti-oxidant genes [18, 33]. Several ARE-dependent genes were then tested, including NADPH quinone oxidoreductase 1 (NQO1), heme oxygenase-1 (HO1) and glutamate cysteine ligase catalytic subunit (GCLC) [34, 35]. Results from quantitative real-time PCR (“qRT-PCR”) assay showed that, as compared to parental (“PAR”) hFOB1. 19 cells, mRNA expressions of NQO1, HO1 and GCLC were significantly increased in cells with miR-455 or Cul3-shRNA (Figure 2A). These results indicated Nrf2 signaling activation. Moreover, H_2_O_2_-induced ROS production was largely attenuated by either miR-455 expression or Cul3-shRNA in hFOB1. 19 cells (Figure 2B). Consequently, H_2_O_2_-induced hFOB1. 19 cell viability loss (MTT OD reduction, Figure 2C), cell death (Trypan blue increase, Figure 2D) and apoptosis (Histone DNA ELISA OD increase, Figure 2E) were also dramatically attenuated after expressing miR-455 or Cul3-shRNA. The microRNA-control (“miRC”) had no significant effect on ARE gene expression (Figure 2A) nor ROS production (Figure 2B). These results indicate that Cul3 knockdown by expressing miR-455 or targeted-shRNA protects hFOB1. 19 cells from H_2_O_2_.

### miR-455 anti-sense induces Cul3 upregulation and Nrf2 degradation

**Figure 3:**
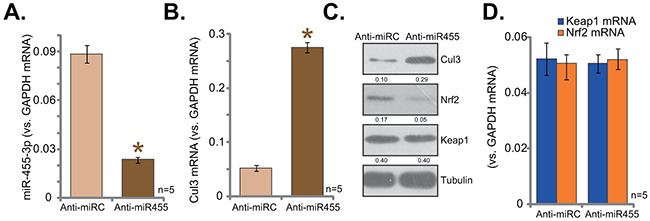
miR-455 anti-sense induces Cul3 upregulation and Nrf2 degradation hFOB1. 19 cells were transfected with miR-455 anti-sense (“Anti-miR-455”, for 5 rounds) or miR anti-sense control (“Anti-miRC”), cells were then subjected to qRT-PCR assay (**A**, **B**, and **D**) and Western blotting assay (**C**) of listed miRNA and genes. Expression of listed proteins was quantified, and was normalized to loading control Tubulin (**C**). Data were shown as mean (n=5) ± standard deviation (SD). *p<0.05 vs. “Anti-miRC” cells. Experiments in this figure were repeated four times, and similar results were obtained.

To further confirm that miR-455 selectively targets Cul3, the miR-455 anti-sense (“Anti-miR-455”) was introduced to hFOB1. 19 cells. As shown in Figure 3A, Anti-miR-455 indeed depleted miR-455 in hFOB1. 19 cells. Consequently, Cul3 mRNA (Figure 3B) and protein (Figure 3C) expressions were upregulated. Nrf2 protein, on the other hand, was degradated (Figure 3C). Nrf2 mRNA, along with Keap1 mRNA, were yet unchanged (Figure 3D). Therefore, Anti-miR-455 depleted miR-455, causing Cul3 upregulation and Nrf2 degradation in hFOB1. 19 cells. These results further confirm that miR-455 selectively targets Cul3 in hFOB1. 19 cells.

### Exogenous over-expression of Cul3 causes Nrf2 degradation

**Figure 4:**
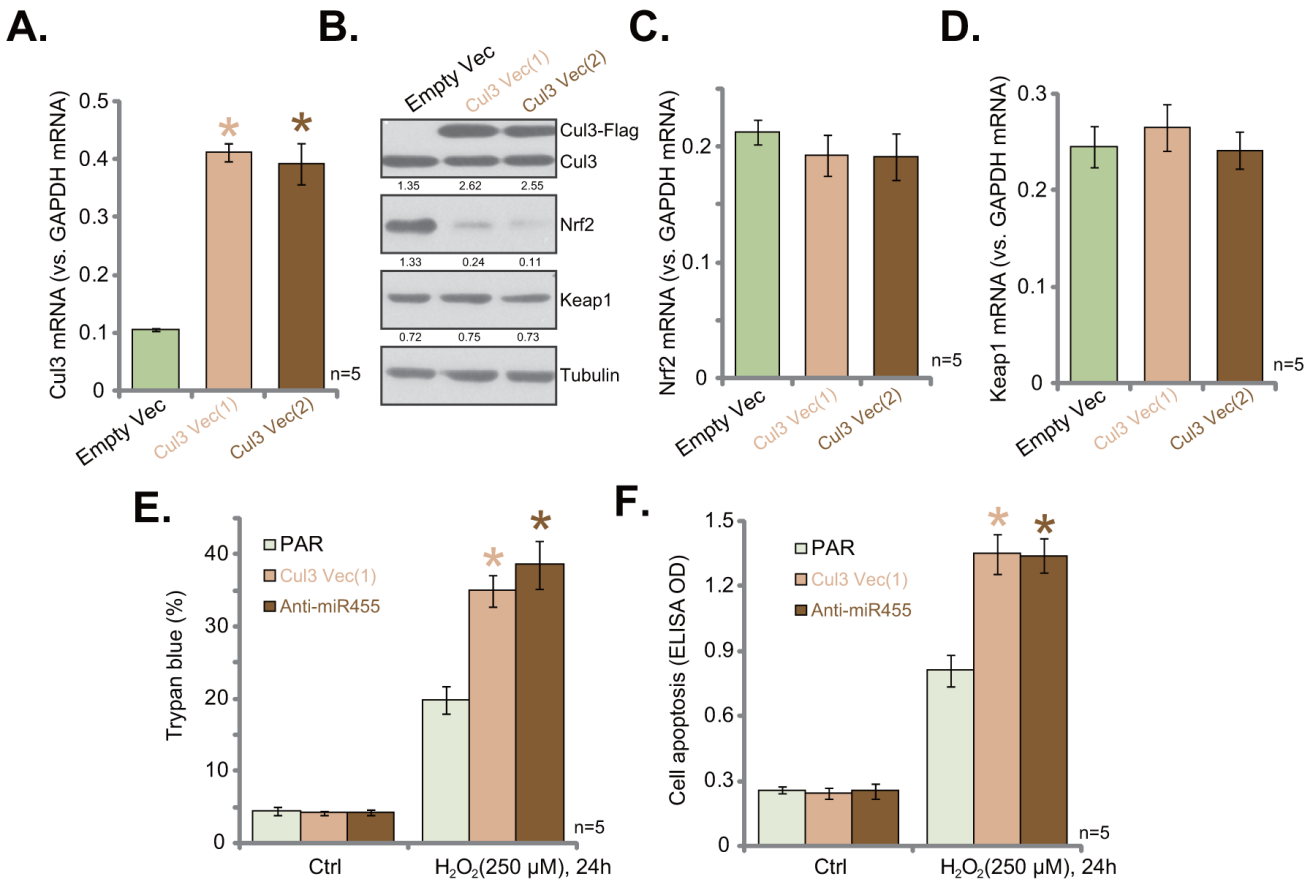
Exogenous over-expression of Cul3 causes Nrf2 degradation Stable hFOB1. 19 cells, expressing Cul3-expressing Vector [two lines, “Vec (1)/(2)”] or empty vector (“Empty Vec”, pSuper-puro-Flag-GFP) were subjected to qRT-PCR assay (**A**, **C** and **D**) and Western blotting assay (**B**) of listed genes. Expression of listed proteins was quantified, and was normalized to loading control Tubulin (**B**). Stable hFOB1. 19 cells, expressing Cul3-expressing Vector [“Vec (1)”], or miR-455 anti-sense (“Anti-miR-455”) as well as the parental control cells (“PAR”) were treated with/out H_2_O_2_ (250 μM) for 24 hours. Cell death and apoptosis were tested by Trypan blue staining assay (**E**) and histone DNA ELISA assay (**F**), respectively. Data were shown as mean (n=5) ± standard deviation (SD). *p<0.05 vs. “Empty Vec” cells (A). *p<0.05 vs. “PAR” cells (**E** and **F**). Experiments in this figure were repeated five times, and similar results were obtained.

Based on the above results, we speculate that Cul3 over-expression should cause Nrf2 protein degradation. To test this hypothesis, the Cul3 expression vector was constructed, and was introduced to hFOB1. 19 cells. Via puromycin selection, two hFOB1. 19 cell lines with the Cul3 construct were established, namely “Cul3 Vec(1)/(2)”. As shown in Figure 4A, Cul3 mRNA level was significantly increased in the two stable lines. Western blotting assay results in Figure 4B confirmed the expression of exogenous Cul3 (Flag-tagged) and endogenous Cul3 in the two lines. Notably, exogenous over-expression of Cul3 indeed led to Nrf2 protein degradation in hFOB1. 19 cells. Nrf2 mRNA (Figure 4C) and Keap1 expression (Figure 4B and 4D) were unchanged with Cul3 over-expression.

Cul3 upregulation and Nrf2 degradation were observed in hFOB1. 19 cells expressing Anti-miR-455 (Figure 3) and Cul3-expressing construct (Figure 4A-4D), we then tested H_2_O_2_ sensitivity in these cells. As compared to the parental control cells (“PAR”), H_2_O_2_ (250 μM)-induced cell death (Figure 4E) and apoptosis (Figure 4F) were dramatically exacerbated in cells expressing Anti-miR-455 or Cul3 vector. These results suggested that Cul3 upregulation induced Nrf2 degradation and facilitated H_2_O_2_-induced killing of hFOB1. 19 cells.

### miR-455-induced Cul3 silence protects primary human osteoblasts from H_2_O_2_

**Figure 5:**
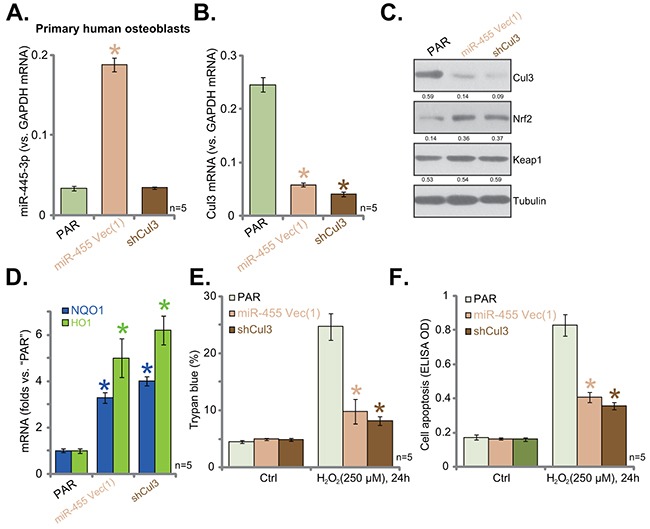
miR-455-induced Cul3 silence protects primary human osteoblasts from H_2_O_2_ Puromycin-selected stable primary human osteoblasts, expressing miRNA-455 Vector [“Vec (1)”] or the Cul3-shRNA (“shCul3”), as well as the parental control cells (“PAR”) were subjected to qRT-PCR assay (**A**, **B** and **D**) and Western blotting assay (**C**) of listed microRNA or genes. Expression of listed proteins was quantified, and was normalized to loading control Tubulin (C). Cells were also treated with/out H_2_O_2_ (250 μM, for 24 hours). Cell death and apoptosis were tested by Trypan blue staining assay (**E**) and histone DNA ELISA assay (**F**), respectively. Data were shown as mean (n=5) ± standard deviation (SD). *p<0.05 vs. “PAR” cells. Experiments in this figure were repeated twice, and similar results were obtained.

The results above indicated that Cul3 silence could protect hFOB1. 19 cells from H_2_O_2_. Next, we tested this hypothesis in primary human cells. Primary-cultured human osteoblasts were constructed with miR-455-expressing vector [“Vec (1)”] or the Cul3-shRNA. Via puromycin selection, stable cells were established. miR-455 (-3p) level was only increased in cells expressing miR-455 vector, but not Cul3-shRNA (Figure 5A). Notably, Cul3 downregulation (Figure 5B and 5C) and Nrf2 protein stabilization (Figure 5C) were observed in cells expressing miR-455 or Cul3-shRNA. Consequently, upregulation of ARE-dependent genes, NQO1 and HO1, was achieved in above mentioned primary cultured human osteoblasts (Figure 5D). More importantly, H_2_O_2_ (250 μM)-induced cell death (Figure 5E) and apoptosis (Figure 5F) were largely inhibited in the primary cells expressing miR-455 or Cul3-shRNA. Thus, we imply that Cul3 silence, by expressing miR-455 or targeted-shRNA, protects primary human osteoblasts from H_2_O_2_.

## DISCUSSION

Nrf2 stabilization and activation induces ARE-dependent transcription of multiple antioxidant defense genes [34, 35], including *NQO1*, *HO1*, *GCLC* [21, 36–38]. Nrf2-ARE signaling has become an attractive target for prevention of human osteoblast injuries. Li *et al*., previously demonstrated that SC79, a novel Akt activator, protected osteoblasts from dexamethasone though activating Akt downstream Nrf2 signaling [39]. Meanwhile, icariside II-induced osteoblast cytoprotection requires Nrf2 activation [40]. Further, cytoprotection of chlorogenic acid against H_2_O_2_-induced oxidative stress in osteoblasts also relies on activation of Nrf2-HO-1 signaling [41]. Nrf2 signaling activation is primarily based on Nrf2 dissociation from its inactive repressor protein Keap1, and the subsequent translocation of Nrf2 to the cell nuclei [21, 36–38]. Many natural and synthetic chemicals have been shown to interfere Nrf2-Keap1 association, causing Nrf2 protein stabilization and activation [28, 29, 31, 42]. Other studies have been able to provoke Nrf2 activation via inhibition, silence, mutation or depletion of Keap1 [28, 29, 31, 42].

Cul3 is a member of the cullin-based ubiquitin ligase family, which is required for Nrf2 degradation [43]. Cul3 forms a complex with Hrt1 and BTB-domain containing proteins, which functions as an E3 ligase to bring Keap1 to ubiquitination and degradation [43]. On the other hand, Cul3 inhibition, silence or mutation will cause inhibition of Nrf2 degradation, and Nrf2 protein stabilization [44]. Recent studies have proposed that miRNA could be a novel and promising strategy to provoke Nrf2 signaling activation (mostly by targeting Keap1) [28, 29]. Very few have focused on miRNA-mediated targeting of Cul3.

Our results here demonstrated that miR-455 is a Cul3-targeting miR in human osteoblasts. Forced-expression of miR-455 in human osteoblasts led to Cul3 degradation, Nrf2 protein stabilization and subsequent transcription of ARE-dependent genes (*NQO1*, *HO1* and *GCLC*). Remarkably, Cul3 silencing by miR-455 expression or targeted-shRNA protected human osteoblasts from H_2_O_2_. On the other hand, miR-455 depletion by miR-455 anti-sense led to Cul3 upregulation and Nrf2 protein degradation, which then exacerbated H_2_O_2_ damages in human osteoblasts. These results together indicate that miR-455 expression could be a novel strategy to provoke Nrf2-ARE signaling activation in human osteoblasts. It will also be interesting to test the *in vivo* function of miR-455 against oxidative-damaged human osteoblasts. Expressions of miR-455 and Cul3 in human osteoporosis and osteonecrosis tissues should also be tested in future studies.

## CONCLUSIONS

Together, our results suggest that miR-455 activates Nrf2 signaling via silencing Cul3, and protects human osteoblasts from oxidative stress.

## MATERIALS AND METHODS

### Reagents

Puromycin was purchased from Sigma Aldrich (St. Louis, MO). All the antibodies were purchased from Cell Signaling Tech (Beverly, MA). Cell culture reagents were obtained from Gibco (Nantong, China).

### Culture of osteoblastic cell line

The hFOB1.19 human osteoblastic cell line [45, 46] was obtained from the Cell Bank of Shanghai Institute of Biological Science (Shanghai, China). Cells were maintained in α-modified essential medium (α-MEM) supplemented with 10% FBS, under 37°C in the presence of 5% CO_2_. Cells were fully differentiated as described [47].

### Primary culture of human osteoblasts

The trabecular bone fragments from healthy donors were minced into small pieces, which were digested by incubation with 5 mg/mL collagenase D (Sigma) for 90 min at 37 °C with agitation. The resulting trabecular bone fragments were further digested with 0.5 mg/mL collagenase D overnight at 37 °C. Cells were then filtered through a 70-μm nylon mesh, and were placed onto the culture flasks with the described medium [48]. Medium was changed three times a week until reaching confluence, and were fully differentiated as described [47]. Primary human osteoblasts were used for further experiments stating at passage 3. The protocols of using human tissues and cells were approved by Ethics Board of Nantong University. Written-informed consent was obtained from each donor.

### Cell viability assay

Human osteoblasts (5000 cells per each well) were initially seeded onto 96-well plates. Following the applied treatment, the MTT dye (20 μL/per well, 5 mg/mL, Sigma) was added to the supernatant for two hours. Afterwards, the optic density (OD) absorbance of MTT at 450 nm was measured by a microplate reader to reflect cell viability.

### Cell death assay

Trypan blue staining assay was performed to test cell death after applied treatment. Cells excluding the dye were considered alive. Trypan blue positive cells were considered dead, and the ratio was recorded using an automatic cell counter.

### Apoptosis quantification by ELISA assay

Thenucleosomal histone-bound DNA fragmentation is the characteristic marker of cell apoptosis, which was examined by the commercial available ELISA kit (Roche, Shanghai, China), using the anti-histone antibody and a secondary anti-DNA antibody. The ELISA OD at 450 nm was tested as the quantitative measurement of cell apoptosis.

### Forced-expression of miR-455

The miR-455 precursor was purchased from RiboBio (Guangzhou, China), which was inserted to the pSuper-GFP-puro vector (Ambion, Shanghai, China) to establish the miR-455-expression vector. Human osteoblasts were transfected with the miR-455 construct or the scramble non-sense microRNA control (“miRC”, Genepharm, Shanghai, China) using the Lipofectamine 2000 reagent (Invitrogen). Stable cells were selected by puromycin (2.5 μg/mL, Sigma) for another 96 hours. Over 95% of stable cells were GFP positive. miR-455 (3p) expression was always verified by the qRT-PCR assay.

### miR-455 anti-sense expression

The hFOB1. 19 osteoblasticcells were transfected with 20 nM of miR-455 anti-sense (“Anti-miR-455”, Ambion, Shanghai, China) by Lipofectamine 2000 (Invitrogen). After two days, cells were split and were transfected with Anti-miR-455 again. This process was repeated for five rounds for a total of 10 days. Expression of miR-455 in the stable cells was examined by qRT-PCR assay. The Ambion miRNA anti-sense negative control (“Anti-miRC”) was transfected to hFOB1. 19 cells as the control cells.

### Western blotting assay

Equivalent amount of total cellular proteins (30 μg per lane) were extracted by a RIPA buffer (Biyuntian, Wuxi, China), and were separated by the 10% SDS gel, prior to transfer onto polyvinylidene difluoride (PVDF) membranes (Millipore, Shanghai, China). The blots were then blocked in 5 % (m/v) milk dissolved in Tris-buffered saline with 0.05 % (w/v) Tween-20 (TBS-T), and were probed with the designated primary and secondary antibodies. The protein signals were visualized under an enhanced chemiluminescence (ECL) system (Amersham Bioscience, Shanghai, China). β-Tubulin (“Tubulin”) was always tested as the loading control. The images were analyzed with Image J software.

### Cul3-shRNA

The lentiviral Cul3-shRNA particles (with GFP-tag) were purchased from Santa Cruz Biotech (sc-35130-V, Nanjing, China). The lentiviral particles (20 μL/mL, per each well) were added to cultured human osteoblasts for 48 hours. Stable cells were again selected by puromycin (2.5 μg/mL, Sigma) for 96 hours. Over 98% of stable cells were GFP positive. Cul3 knockdown in the stable cells was verified by Western blotting assay and qRT-PCR assay. For the control cells, the lentiviral scramble control shRNA particles (Santa Cruz Biotech) were added.

### Exogenous Cul3 over-expression

The full-length human *Cul3 cDNA* was synthesized by Genepharm (Shanghai, China), which was inserted to the pSuper-puro-GFP-Flag vector (Addgene, Shanghai, China). Lipofectamine 2000 was applied to transfect the Cul3 construct to human osteoblasts. Puromycin (2.5 μg/mL, Sigma) was added to select stable cells for 96 hours. Over 98% of cells were GFP positive. Expression of endogenous and exogenous (Flag-tagged) Cul3 in the stable cells was verified by Western blotting assay and qRT-PCR assay. The empty pSuper-puro-GFP-Flag vector was transfected to the control cells.

### Quantitative RT-PCR

Trizol reagents (Invitrogen) were utilized to extract total cellular RNA, and the High Capacity cDNA Reverse Transcription Kit was applied to synthesize cDNA from 0.5 μg mRNA per treatment. Quantitative real-time PCR (“qRT-PCR”) assay was performed by the Power SYBR Green RT-PCR Reagents Kit using the ABI-7500 system [49]. We utilized 2^ΔΔCt^ method to yield relative *mRNA* fold expression (as compare to GAPDH mRNA). mRNA primers for *HO-1* and *GCLC* were described previously [50]. mRNA primers for *Nrf2*, *Keap1*, *Cul3*, *NQO1* and GAPDH were described early [51]. miR-455 (-3p) expression was tested via the TaqMan microRNA assay [52] (Applied Biosystems, Shanghai, China), from 5 ng of total RNA [53].

### ROS assay

As described previously [16, 40, 54–56], we utilized the dichloro-dihydro-fluorescein diacetate (DCFH-DA) fluorescent dye (Invitrogen) assay to determine the intracellular ROS intensity. Briefly, after the applied treatment, DCFH-DA dye (5.0 μg/mL) was added to cells, followed by three-founds wash in warm PBS. Afterwards, the DCFH-DA fluorescence OD, reflecting the relative ROS intensity, was examined by a Fluorescence/Multi-Detection Microplate Reader (Synergy 2, BioTek).

### Statistics

Data were expressed as the mean ± SD [45, 57]. Comparisons between groups were performed via one-way ANOVA and then Student-Newman-Keuls test (SPSS 18.0). The *p* values < 0.05 were considered statistically significant.
